# A Highly Permissive K-C4 Cell Clone for Genotype II African Swine Fever Virus Reveals Passage-Dependent Hemadsorption Changes Associated with EP402R Deletion

**DOI:** 10.3390/vetsci13101024

**Published:** 2026-09-28

**Authors:** Eun-Gyeong Lee, Ju-Hun Kim, Sarah Lim, Sung-Hyun Moon, Hyunji Gu, Jun Young Park, Dae-Min Kim, Gun-Hee Lee, Ho-Seong Cho, Kyeong-Man Kim, Dongseob Tark

**Affiliations:** 1 Laboratory for Infectious Disease Prevention, Korea Zoonosis Research Institute, Jeonbuk National University, Iksan 54531, Republic of Korea; eunlee@jbnu.ac.kr (E.-G.L.); sarah31217@jbnu.ac.kr (S.L.); guhji104@jbnu.ac.kr (H.G.); tennis098@naver.com (J.Y.P.); daeminkk@gmail.com (D.-M.K.); ghlee80@jbnu.ac.kr (G.-H.L.); 2 Department of Pharmacology, College of Pharmacy, Chonnam National University, Gwangju 61186, Republic of Korea; khjh99@hanmail.net; 3 College of Veterinary Medicine and Bio-Safety Research Institute, Jeonbuk National University, Iksan 54596, Republic of Korea; chunsu17@naver.com (S.-H.M.); hscho@jbnu.ac.kr (H.-S.C.)

**Keywords:** African swine fever virus, genotype II, COS-1 cells, cell cloning, cell culture adaptation, hemadsorption, *EP402R*

## Abstract

African swine fever virus (ASFV) research commonly relies on primary porcine alveolar macrophages (PAMs), whose limited availability and variability hinder standardized virus isolation and propagation. This study aimed to establish a continuous cell line highly susceptible to genotype II ASFV and characterize phenotypic and genomic changes during serial passage. Thirty COS-1-derived clones were screened after infection with Korea/Pig/PAJU1/2019 using quantitative real-time polymerase chain reaction, immunofluorescence, and hemadsorption assays. Selected clones underwent serial passage, and viruses from passages 5 and 10 in the most susceptible clone were analyzed by whole-genome sequencing. K-C4 supported stable ASFV replication, with a mean viral genome level of 8.80 ± 0.55 log_10_ copies/mL, compared with 7.38 ± 0.90 log_10_ copies/mL in PAMs. Productive infection was confirmed by p72-specific immunofluorescence. The parental and passage 1 viruses retained hemadsorption, whereas the passage 5 virus lacked detectable hemadsorption despite continued replication, and hemadsorption reappeared by passage 10. Whole-genome analysis identified passage-associated deletions of differing extent within EP402R, encoding the hemadsorption-associated CD2-like viral protein (CD2v), which corresponded with the hemadsorption phenotype at each passage. These findings establish K-C4 as a highly permissive platform for genotype II ASFV propagation and characterization while emphasizing the need to monitor phenotypic and genomic stability during serial passage.

## Simple Summary

African swine fever is a deadly pig disease that causes major economic losses, but research is hindered because the virus is grown in pig lung immune cells that are difficult to obtain and vary between animals. We aimed to develop a reliable cell line, a population of cells that can be maintained in the laboratory, for growing the virus. We tested 30 populations, each grown from a single cell, and selected K-C4 because it supported efficient and consistent virus growth. Under our conditions, K-C4 produced viral genetic material at levels comparable to or higher than pig lung macrophage cells and continued to support growth through repeated transfers. However, after repeated transfers, the virus temporarily lost its ability to make pig red blood cells attach to infected cells, and this ability returned after further passage. Analysis of the virus’s genetic material showed that these changes tracked with the size of a partial deletion in a viral gene that produces the protein responsible for attachment. K-C4 could provide a consistent and practical system for growing and studying African swine fever virus and supporting disease testing and vaccine research. The findings also show that viruses repeatedly grown in laboratory cells must be monitored for genetic changes and altered behavior.

## 1. Introduction

African swine fever (ASF) is a severe hemorrhagic disease of domestic pigs and wild boar that imposes substantial health and economic burdens on swine production worldwide. The causative agent, African swine fever virus (ASFV), is a large enveloped double-stranded DNA virus of the family *Asfarviridae*. Its genome is approximately 170–190 kbp in length and is genetically diverse, with p72-based classification using sequence variation in the *B646L* gene historically distinguishing at least 24 genotypes [1,2]. Following its emergence in the Caucasus in 2007, genotype II ASFV expanded across large areas of Europe and Asia. In the Republic of Korea, where ASF was first confirmed in 2019, infections have subsequently been documented in both domestic pigs and wild boar, complicating sustained surveillance and disease-control efforts [3,4]. Consequently, effective ASF management has relied heavily on early detection, stringent biosecurity, movement control, and rapid implementation of outbreak-eradication measures [5]. Isolation and propagation of ASFV are essential for studies of viral pathogenesis, diagnostics, vaccine development, and antiviral evaluation. Macrophages are the primary target cells of ASFV during natural infection [6]. Accordingly, primary porcine alveolar macrophages (PAMs) have traditionally served as the reference cell system for ASFV isolation and propagation [7,8]. However, routine use of PAMs is limited by the need for donor animals, batch-to-batch variability, labor-intensive preparation procedures, and difficulties in standardizing large-scale experimental applications [7,8,9]. These practical constraints have prompted the evaluation of continuous cell substrates as alternatives for ASFV propagation [7]. However, the permissiveness of candidate porcine and non-porcine cell lines varies considerably and is influenced by both the cellular background and the ASFV isolate examined [8,9,10]. A continuous cell system that combines high ASFV permissiveness with reproducible growth characteristics would therefore facilitate virus propagation and a range of downstream virological applications. An additional consideration, however, is that repeated propagation in an alternative cellular environment may alter the genetic composition of the virus population. Indeed, adaptation of ASFV to non-macrophage cell lines has been associated with passage-dependent deletions and genomic rearrangements involving loci related to replication, host interaction, and virulence [11,12,13]. The resulting sequence changes can be accompanied by altered viral phenotypes, providing a basis for examining how ASFV populations respond to sustained growth in heterologous cells. Hemadsorption (HAD), the binding of erythrocytes to ASFV-infected cells, is largely mediated by CD2v, the viral protein encoded by *EP402R* [14]. Accordingly, disruption or loss of functional *EP402R* sequences can alter the HAD phenotype, making this locus particularly informative when phenotypic changes arise during cell-culture adaptation [14,15]. Previous studies have shown that alterations affecting *EP402R* may result in changes in hemadsorption activity, highlighting the importance of this genomic region in ASFV biology [14,15]. We therefore generated clonal populations from COS-1 cells and selected K-C4 for detailed evaluation of its ability to propagate the genotype II isolate Korea/Pig/PAJU1/2019. We further examined whether serial passage in K-C4 was accompanied by changes in HAD activity and by corresponding sequence alterations within the *EP402R* region.

## 2. Materials and Methods

### 2.1. Virus and Cells

The African swine fever virus (ASFV) isolate Korea/Pig/PAJU1/2019 (ASFV PAJU1; GenBank accession no. MT748042.2), a highly virulent genotype II strain, was obtained from the Animal and Plant Quarantine Agency (APQA, Republic of Korea). Virus stocks were initially propagated in porcine alveolar macrophages (PAMs) and subsequently used for infection experiments and serial passage studies. Porcine alveolar macrophages (PAMs) were used as the reference permissive cell system for ASFV propagation and comparison of viral replication efficiency. The African green monkey kidney cell line COS-1 was obtained from the Korean Cell Line Bank (KCLB; cell line no. 21650, Seoul, Republic of Korea). The K-C4 cell line used in this study was established through clonal selection of COS-1 cells by limiting dilution and was subsequently evaluated for susceptibility to ASFV infection. All experiments involving infectious African swine fever virus (ASFV) were performed in accordance with the institutional biosafety regulations and were approved by the Institutional Biosafety Committee (IBC) of Jeonbuk National University (IBC approval no. JBNU2023-01-002).

### 2.2. Preparation and Culture of Porcine Alveolar Macrophages (PAMs)

Porcine alveolar macrophages (PAMs) were obtained from specific-pathogen-free (SPF) Yucatan miniature pigs supplied by Optipharm Inc. (Cheongju, Republic of Korea). Following euthanasia with Zoletil (Virbac, Carros, France) and xylazine hydrochloride (Rompun; Bayer, Leverkusen, Germany), lungs were aseptically collected and lavaged with phosphate-buffered saline (PBS) supplemented with 1% penicillin–streptomycin (Gibco, Thermo Fisher Scientific, Waltham, MA, USA). The recovered lavage fluid was filtered through sterile gauze to remove tissue debris and centrifuged to obtain PAMs [16]. The harvested cells were cultured in RPMI 1640 medium (Gibco, Thermo Fisher Scientific, Waltham, MA, USA) supplemented with 10% fetal bovine serum (FBS), 1% penicillin–streptomycin, 1% minimum essential medium non-essential amino acids (MEM NEAA), 1% sodium pyruvate, 1% 4-(2-hydroxyethyl)-1-piperazineethanesulfonic acid (HEPES) buffer (all from Gibco), and 0.1% 2-mercaptoethanol (Sigma-Aldrich, St. Louis, MO, USA). Cells were maintained at 37 °C in a humidified incubator with 5% CO_2_. All animal procedures were approved by the Institutional Animal Care and Use Committee (IACUC) of Jeonbuk National University (IACUC approval no. NON2022-08-016).

### 2.3. Maintenance of COS-1 and K-C4 Cells

COS-1 and K-C4 cells were maintained in Dulbecco’s Modified Eagle Medium (DMEM; Gibco, Thermo Fisher Scientific, Waltham, MA, USA) supplemented with 10% fetal bovine serum (FBS) and 1% penicillin–streptomycin. Cells were cultured at 37 °C in a humidified incubator with 5% CO_2_.

### 2.4. Cell Cloning by Limiting Dilution

To establish clonal cell populations, COS-1 cells were subjected to limiting dilution. Cells were serially diluted in growth medium consisting of Dulbecco’s Modified Eagle Medium (DMEM) supplemented with 10% fetal bovine serum (FBS) and 1% penicillin–streptomycin and seeded into 96-well plates at approximately one cell per well. Plates were incubated at 37 °C in a humidified atmosphere containing 5% CO_2_. Individual wells were monitored microscopically, and wells containing single-cell-derived colonies were selected for expansion. Following growth to near confluence, clones were transferred to 12-well plates and further expanded. A total of 30 independent COS-1-derived clones were established. Each clone was subsequently divided into duplicate cultures. One culture was infected with ASFV, whereas the corresponding duplicate culture was maintained for further propagation. Viral replication in infected cultures was evaluated by real-time PCR, and clones exhibiting high susceptibility to ASFV infection were selected for subsequent characterization and analysis.

### 2.5. Quantitative Real-Time Polymerase Chain Reaction (qPCR)

Cells infected with ASFV were harvested at 6 days post-infection (dpi), and viral DNA was extracted from cell lysates using the DNeasy Blood & Tissue Kit (Qiagen, Hilden, Germany) according to the manufacturer’s instructions. Detection of the ASFV genome was performed using the VetMAX™ African Swine Fever Virus Detection Kit (Thermo Fisher Scientific, Waltham, MA, USA) on a QuantStudio™ 3 Real-Time PCR System (Thermo Fisher Scientific) following the manufacturer’s protocol. For serial-passage experiments, ASFV DNA in cell-culture supernatants was quantified by qPCR targeting the p72 region to monitor virus production across successive passages. ASFV genome concentrations were calculated from a calibration curve generated with 10-fold serial dilutions of quantified viral DNA, following the general DNA-copy quantification approach described previously [17], and are reported as log_10_ genomic copies/mL. Data analysis was performed using GraphPad Prism software (version 8.0, GraphPad Software, San Diego, CA, USA).

### 2.6. Hemadsorption Assay (HAD)

To evaluate changes in hemadsorption activity during serial passage of ASFV in K-C4 cells, porcine alveolar macrophages (PAMs) and K-C4 cells were seeded in 6-well plates and infected with the parental ASFV PAJU1 strain, ASFV-K-C4 passage 1 (P1), ASFV-K-C4 passage 5 (P5) and ASFV-K-C4 passage 10 (P10). Following virus inoculation, culture medium containing 1% porcine red blood cells (RBCs) was added to each well. Cells were incubated at 37 °C in a humidified atmosphere containing 5% CO_2_ and examined daily by light microscopy. Hemadsorption activity was assessed based on the formation of characteristic erythrocyte rosettes surrounding infected cells. Representative images were acquired at 4 days post-infection (dpi) and compared among parental and serially passaged viruses to evaluate phenotypic changes associated with serial passage in K-C4 cells.

### 2.7. Immunofluorescence Assay (IFA)

To confirm ASFV infection in K-C4 cells, porcine alveolar macrophages (PAMs) and K-C4 cells were seeded in 6-well plates and infected with ASFV. At 4 days post-infection (dpi), cells were washed three times with phosphate-buffered saline (PBS) and fixed with 4% paraformaldehyde. Fixed cells were incubated with a recombinant anti-ASFV p72 monoclonal antibody (Creative Biolabs, Shirley, NY, USA), followed by an Alexa Fluor^®^ 488-conjugated anti-mouse IgG secondary antibody (Cell Signaling Technology, Danvers, MA, USA). Fluorescence signals were visualized using a CELENA^®^ X High Content Imaging System (Logos Biosystems, Anyang, Republic of Korea), and images were analyzed using CELENA^®^ X Cell Analyzer software (version 1.6.0).

### 2.8. Next-Generation Sequencing (NGS)

To investigate genomic changes occurring during serial passage in K-C4 cells, viral DNA was extracted from ASFV-K-C4 passage 5 (P5) and passage 10 (P10) virus stocks. Sequencing libraries were prepared using the CELEMICS NGS Library Prep and Target Enrichment workflow (Celemics, Seoul, Republic of Korea) and sequenced on an Illumina iSeq 100 platform (Illumina, San Diego, CA, USA) using the iSeq 100 i1 Reagent Kit v2. Raw sequence reads were subjected to quality trimming and de novo assembly using CLC Genomics Workbench (version 24.0.3; Qiagen, Hilden, Germany). Assembled contigs were subsequently mapped to the ASFV Georgia 2007/1 reference genome (FR682468.2) using the same software. Genome coverage, consensus sequence length, and genomic variations were analyzed using CLC Genomics Workbench. Whole-genome comparison maps were generated with Proksee [18] by aligning the assembled sequences with ASFV Georgia 2007/1 (FR682468.2) and Korea/Pig/PAJU1/2019 (MT748042.2), allowing passage-associated genomic differences to be visualized. Sequence differences within the *EP402R* (CD2v) region were examined by multiple sequence alignment and displayed with the NCBI Multiple Sequence Alignment Viewer (version 1.26.0; https://www.ncbi.nlm.nih.gov/projects/msaviewer/ (accessed on 21 September 2023)).

### 2.9. Statistical Analysis

Graphs and statistical analyses were performed using GraphPad Prism software (version 8.0.1, San Diego, CA, USA). Viral genome copy values obtained from K-C4 cells and PAMs were compared using an unpaired, two-tailed Student’s *t*-test. To evaluate the overall replication efficiency between the two cell types, the statistical test was applied to the aggregate data spanning all 10 evaluated passages. Each passage was performed in duplicate, resulting in a total of 20 observations per cell type (n = 20 for both K-C4 and PAM). Statistical significance was defined as *p* < 0.05.

## 3. Results

### 3.1. Establishment and Selection of an ASFV-Susceptible K-C4 Cell Clone

To identify COS-1-derived cell clones with enhanced susceptibility to ASFV infection, a total of 30 independent clones were generated by limiting dilution and expanded for screening (Figure 1). Following infection with ASFV PAJU1, viral replication in each clone was evaluated by quantitative real-time PCR (qPCR) at 6 days post-infection (dpi) (Figure 2A). Most clones supported detectable ASFV replication; however, differences in viral genome levels were observed among individual clones. Based on the initial screening results, three clones (C2, C4, and C9) exhibiting relatively high levels of ASFV replication were selected for further evaluation. To assess their ability to support sustained virus propagation, ASFV was serially passaged in each clone, and viral replication was monitored by qPCR over five consecutive passages (Figure 2B). All three clones remained permissive to ASFV infection throughout the passage series. Among them, clone C4 consistently exhibited higher viral genome copy numbers across serial passages, indicating stable support of viral replication.

Based on these findings, clone C4 was selected for further characterization and designated K-C4.

### 3.2. K-C4 Cells Support Efficient Replication of Genotype II ASFV

To further evaluate the ability of K-C4 cells to support ASFV replication, viral propagation was compared with that observed in primary porcine alveolar macrophages (PAMs), which are widely used for ASFV isolation and propagation. ASFV replication was maintained in K-C4 cells through at least six serial passages, with Ct values remaining below 20 and viral genome levels reaching approximately 8.75 log_10_ copies (Figure 2C). Across serial passages, K-C4 cells consistently supported high levels of ASFV replication, with an average Ct value of 17.95 ± 1.86 and an average viral genome level of 8.8 ± 0.55 log_10_ copies. In comparison, ASFV replication in PAMs yielded an average Ct value of 22.7 ± 3.01 and 7.38 ± 0.90 log_10_ viral copies. These findings demonstrated that K-C4 cells supported efficient replication of the ASFV PAJU1 strain under the experimental conditions used in this study. To confirm productive infection of K-C4 cells, ASFV-infected cultures were examined by immunofluorescence assay (IFA) using an antibody specific for the ASFV major capsid protein p72. Strong p72-specific fluorescence was detected in infected K-C4 cells and was comparable to that observed in infected PAMs, whereas no specific fluorescence signal was detected in mock-infected controls (Figure 3). These observations confirmed productive ASFV infection in K-C4 cells and supported the qPCR-based replication data.

### 3.3. Passage-Dependent Loss and Reappearance of Hemadsorption During Serial Passage of ASFV in K-C4 Cells

To assess passage-associated phenotypic changes, the parental ASFV PAJU1 isolate and viruses collected after 1, 5, and 10 passages in K-C4 cells (P1, P5, and P10, respectively) were inoculated into PAMs and K-C4 cells. Cytopathic effects (CPEs) were observed in all virus-infected cultures (Figure 4). At 4 days post-infection (dpi), hemadsorption (HAD) was evaluated based on the formation of characteristic erythrocyte rosettes. HAD rosettes were observed in both cell types infected with the parental virus or P1. In contrast, no detectable HAD rosettes were observed in P5-infected cultures, despite the presence of CPEs. HAD rosettes reappeared in both PAMs and K-C4 cells infected with P10, indicating that the HAD-negative phenotype observed at P5 was not maintained at P10. To investigate genomic changes potentially associated with the contrasting HAD phenotypes of P5 and P10, the two passaged viruses were subsequently subjected to whole-genome sequencing.

### 3.4. Genomic Alterations Associated with Passage-Dependent Changes in Hemadsorption in K-C4-Passaged ASFV

To investigate genomic changes associated with the passage-dependent alterations in hemadsorption (HAD), ASFV-K-C4 passage 5 (P5) and passage 10 (P10) were subjected to whole-genome sequencing and compared with the parental ASFV PAJU1 isolate and the ASFV Georgia 2007/1 reference genome. Sequencing and genome assembly metrics are summarized in Table 1. A total of 1,267,148 reads were obtained for P5, of which 1,165,008 (91.94%) mapped to the Georgia 2007/1 reference genome, resulting in a mean coverage depth of 797.6×. For P10, 772,655 reads were obtained, of which 463,580 (60.00%) mapped to the reference genome, with a mean coverage depth of 302.5×. The lower proportion of reads mapping to the viral reference at P10, relative to P5, is consistent with a higher relative abundance of host-derived (K-C4 cell) genomic DNA in the P10 extract, likely attributable to passage-dependent variation in viral yield per cell or the extent of cytolysis at the time of harvest rather than to sequencing artefacts; the mean coverage depth of 302.5× at P10 nonetheless remained well above the threshold generally accepted as sufficient for reliable consensus genome assembly and accurate variant detection. The consensus genome lengths of P5 and P10 were 189,129 and 190,187 bp, respectively; thus, the P10 consensus genome was 1058 bp longer than that of P5 (Table 1). Comparative whole-genome analysis identified passage-associated sequence differences in both coding and non-coding regions, including alterations affecting the *MGF100-1R* and *EP402R* loci (Figure 5A). Relative to the parental ASFV PAJU1 and Georgia 2007/1 genomes, deletions within the *EP402R* coding region were detected in both P5 and P10. Based on the Georgia 2007/1 reference genome coordinates, P5 contained a 1032 bp deletion spanning nucleotides 74,969–76,000, whereas P10 contained a substantially shorter 55 bp deletion spanning nucleotides 74,969–75,023. Thus, the deletion interval in P10 was 977 bp shorter than that in P5. The P5 deletion encompassed most of the *EP402R* coding sequence, whereas the substantially smaller deletion in P10 left a much larger portion of *EP402R* represented in the consensus genome (Figure 5B). The reduction in the extent of the *EP402R* deletion at P10 coincided with the reappearance of HAD rosettes, whereas the extensive deletion observed at P5 was associated with the absence of detectable HAD activity. Together, these findings support an association between the extent of the *EP402R* deletion and the passage-dependent HAD phenotype. The change in the consensus sequence profile between P5 and P10 is consistent with a shift in the composition of the viral population during serial passage. However, consensus whole-genome sequencing alone cannot determine whether this change resulted from the selection of pre-existing viral variants, recombination between coexisting viral genomes, or a combination of both processes. Biological cloning followed by clone-specific genotyping and phenotypic characterization will therefore be required to isolate individual *EP402R*-deleted variants and establish a direct genotype–phenotype relationship.

## 4. Discussion

Although PAMs are highly permissive to ASFV, their dependence on donor animals and preparation variability complicates routine standardization, creating a continuing need for reproducible continuous-cell alternatives. In the present study, we established a COS-1-derived cell clone, designated K-C4, and demonstrated that it supports efficient replication of the genotype II ASFV isolate Korea/Pig/PAJU1/2019. Screening of 30 independently derived COS-1 clones identified K-C4 as the most susceptible clone, consistently supporting viral replication through serial passages. Quantitative PCR analysis demonstrated that ASFV replication in K-C4 cells was comparable to, and under the experimental conditions used in this study frequently exceeded, that observed in PAMs. In addition, productive infection was confirmed by p72-specific immunofluorescence. Together, the replication and p72-expression data show that K-C4 can sustain productive infection with Korea/Pig/PAJU1/2019 and perform as a highly permissive continuous-cell substrate under the conditions examined here [7,8,9]. ASFV replication has been investigated in several continuous substrates, including COS-1, Vero, WSL, and immortalized porcine macrophage-derived lines [7,9,10,11], but their permissiveness differs according to both the virus isolate and the cellular background [7,9]. Earlier work established that COS-1 cells can support selected ASFV isolates and can be used for plaque-based virological analyses [10]. The present results add a clonal-selection perspective, showing that individual subpopulations derived from COS-1 can differ markedly in their permissiveness to ASFV. The ability of K-C4 cells to maintain efficient replication during serial passages suggests that cellular heterogeneity within established cell lines may influence ASFV permissiveness and that targeted clonal selection represents a practical strategy for developing improved ASFV culture systems [7,9]. A striking passage-dependent change in HAD phenotype was observed during propagation in K-C4 cells: HAD was retained by the parental virus and P1, was undetectable at P5, and reappeared at P10. Thus, the phenotype did not decline progressively with passage but instead changed in association with the viral population present at different passage levels. Whole-genome analysis showed that the HAD-negative P5 population contained an extensive deletion within *EP402R*, whereas P10, in which HAD reappeared, retained a substantially larger portion of this locus. Because *EP402R* encodes CD2v, a major determinant of erythrocyte binding in ASFV-infected cells [14,15], the correspondence between the extent of *EP402R* sequence loss and HAD status supports a genotype–phenotype association. However, the present consensus-sequence data do not establish that the *EP402R* alteration alone caused the observed phenotypic transition. Because the sequencing libraries were prepared using a target-enrichment approach, in which capture efficiency can vary with the size and structural continuity of the target region, raw NGS read counts for the 1032 bp and 55 bp *EP402R* deletion variants at P5 and P10 could not be reliably converted into quantitative subpopulation allele frequencies. Determining the co-existence ratio of these variants across passages will require a dedicated quantification strategy—such as capture-bias-calibrated shotgun sequencing or biological cloning with clone-specific genotyping—which is beyond the scope of the present study. Notably, the P5 and P10 deletions share an identical start coordinate (nt 74,969), and both consensus sequences were derived at substantial coverage depth (797.6× and 302.5×, respectively) using a de novo assembly-based pipeline; together with the independent concordance between the reduced deletion size at P10 and the reappearance of HAD activity, these features argue against a sequencing or assembly artefact and instead support a genuine, passage-associated change within the *EP402R* locus. This change in the consensus sequence profile between P5 and P10 is consistent with a shift in the composition of the viral population during serial passage. Passage-associated sequence variation in K-C4 is consistent with earlier observations that ASFV adaptation to continuous cells can reshape portions of the viral genome [11,12,13]. Extensive genome modification was reported during Vero-cell adaptation of a genotype II isolate by Krug et al. [11], while studies using other monkey kidney-derived systems likewise documented deletions or rearrangements arising during cell-culture adaptation [12,13]. Although the biological consequences of the *EP402R* deletion identified in the present study require further investigation, our findings indicate that serial propagation of ASFV in K-C4 cells may be accompanied by measurable genomic and phenotypic changes. The passage-dependent changes observed here also emphasize that phenotypic characterization of ASFV propagated in continuous cells should be accompanied by genomic monitoring, particularly when viruses are maintained over multiple passages. K-C4 may therefore be useful not only for propagation but also for examining how ASFV populations change during adaptation to a continuous cellular environment. The substantial genetic and geographic diversity of ASFV documented across Africa, Europe, and Asia [19,20,21,22,23,24] is also relevant to the interpretation of our findings, because only one genotype II isolate was examined in this study. Although genotype II ASFV is widely distributed across Europe and Asia, including the Republic of Korea [3,4,21,22,24], the performance of K-C4 should be evaluated with additional isolates representing distinct genomic backgrounds before its applicability can be generalized beyond Korea/Pig/PAJU1/2019. Longer-term passage studies should therefore determine whether the genomic changes observed here recur reproducibly and clarify how alterations within *EP402R* relate to changes in HAD phenotype during K-C4 adaptation.

## 5. Conclusions

In this study, we established a highly ASFV-susceptible cell clone, designated K-C4, through clonal selection of COS-1 cells and demonstrated its capacity to efficiently support replication of the genotype II ASFV isolate Korea/Pig/PAJU1/2019. Among the 30 COS-1-derived clones evaluated, K-C4 exhibited the highest level of susceptibility and maintained productive viral replication during serial passages. Quantitative real-time PCR and immunofluorescence analyses confirmed that K-C4 cells supported efficient ASFV replication under the experimental conditions used in this study. A notable finding was a passage-dependent change in hemadsorption (HAD) activity during serial passage of ASFV in K-C4 cells: HAD activity, present in the parental virus and at passage 1, was lost by passage 5 and subsequently reappeared by passage 10. Whole-genome sequencing revealed that this phenotypic change was accompanied by partial deletions of differing extent within the *EP402R* coding region, with a more extensive deletion at passage 5 and a smaller residual deletion at passage 10. The correspondence between the extent of *EP402R* deletion and HAD status at each passage suggests that serial passage in K-C4 cells may be accompanied by measurable phenotypic and genomic changes during in vitro propagation. Collectively, these findings demonstrate that K-C4 is a highly permissive cell line for ASFV propagation and characterization and provide new insights into viral changes that may arise during prolonged passage in continuous cell culture systems. The K-C4 cell line may therefore serve as a useful platform for future studies on ASFV biology, virus–host interactions, and cell culture-based virus propagation.

## Figures and Tables

**Figure 1:**
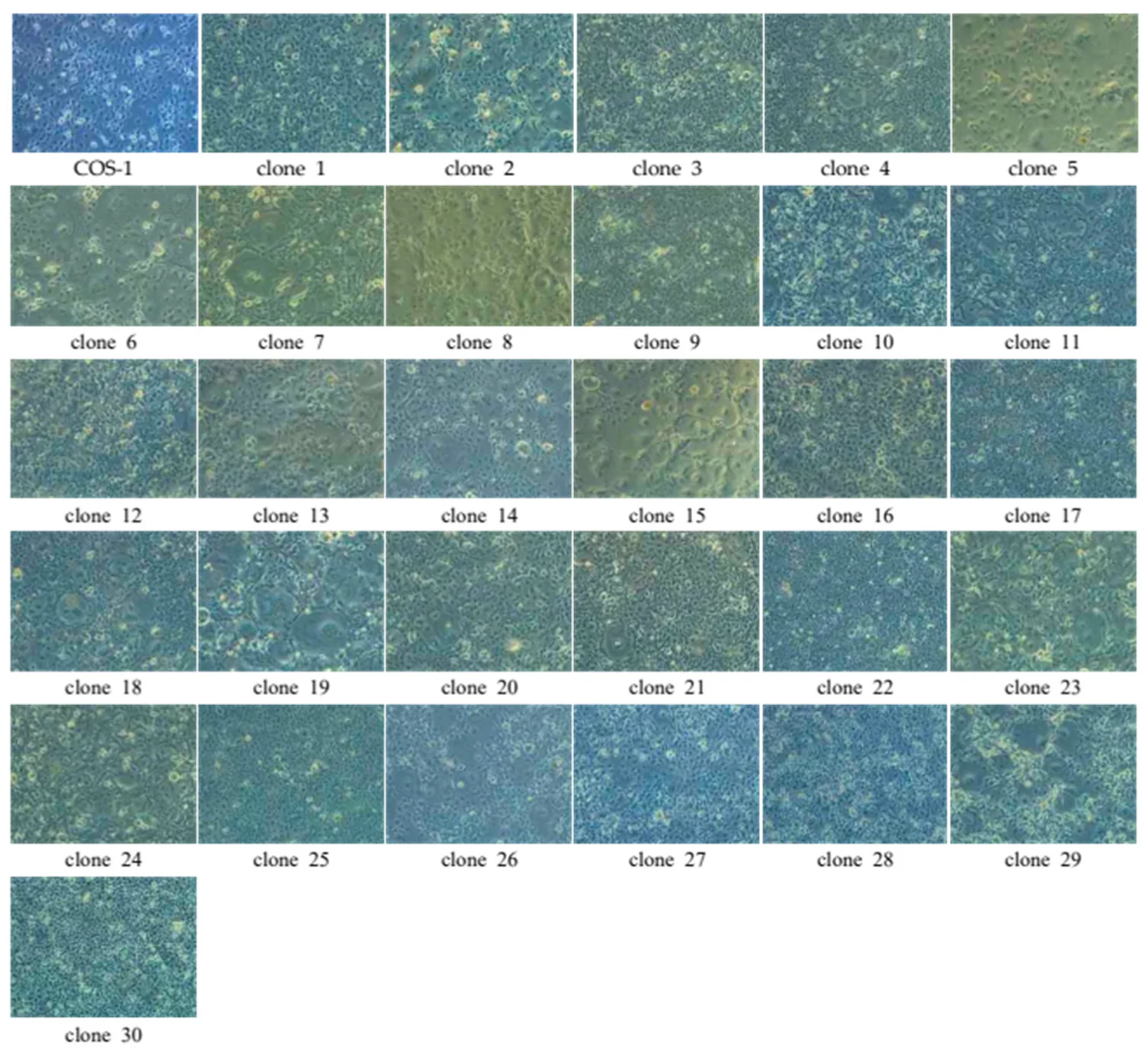
Morphological characteristics of COS-1-derived cell clones generated by limiting dilution. A total of 30 independent cell clones were established from the parental COS-1 cell line by limiting dilution. Representative phase-contrast microscopic images of each clone following expansion in culture are shown. Morphological differences among individual clones were evaluated prior to ASFV susceptibility screening. Images were acquired at 100× magnification.

**Figure 2:**
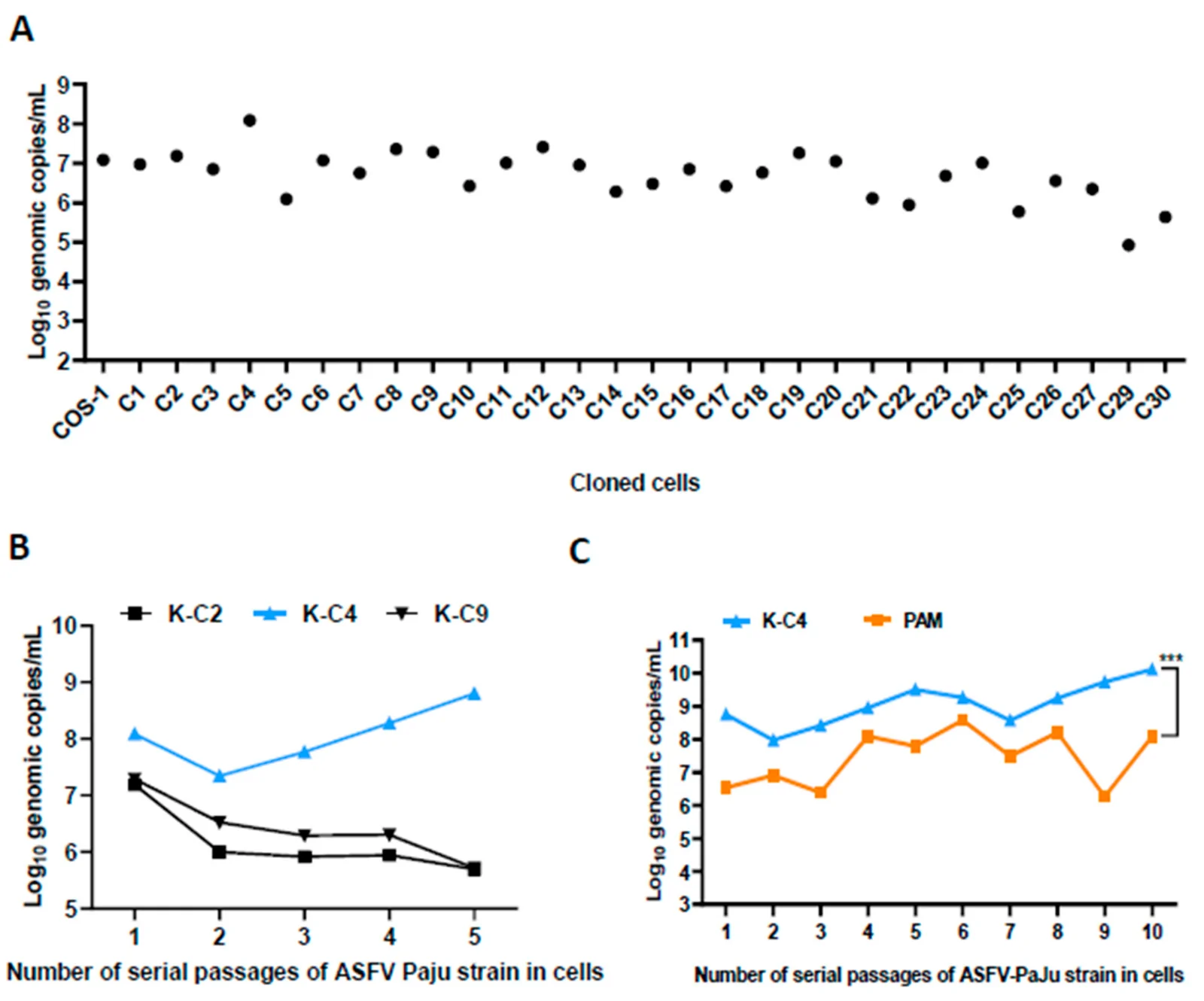
Screening and selection of ASFV-susceptible COS-1-derived cell clones. (**A**) Quantitative real-time PCR (qPCR) analysis of ASFV genome levels in 30 COS-1-derived clones following infection with ASFV Korea/Pig/PAJU1/2019. Cells were harvested at 6 days post-infection (dpi), and ASFV genome levels were determined using the VetMAX™ ASFV Detection Kit and are expressed as log_10_ genomic copies/mL. (**B**) Evaluation of viral replication during five consecutive passages in selected clones (C2, C4, and C9). Virus harvested from each passage was used for subsequent infection, and ASFV genome levels were quantified by qPCR and are presented as log_10_ genomic copies/mL. (**C**) Comparison of ASFV replication efficiency between K-C4 cells and primary porcine alveolar macrophages (PAMs) during serial passage. Viral genome copy numbers were determined by qPCR and expressed as log_10_ genome copies. K-C4 cells consistently supported efficient ASFV replication throughout serial passages. Statistical comparison between K-C4 and PAM groups was performed using an unpaired, two-tailed Student’s *t*-test applied to the aggregate data of 10 continuous passages. Each passage was evaluated in duplicate, yielding 20 independent observations per group (n = 20). *** *p* < 0.001.

**Figure 3:**
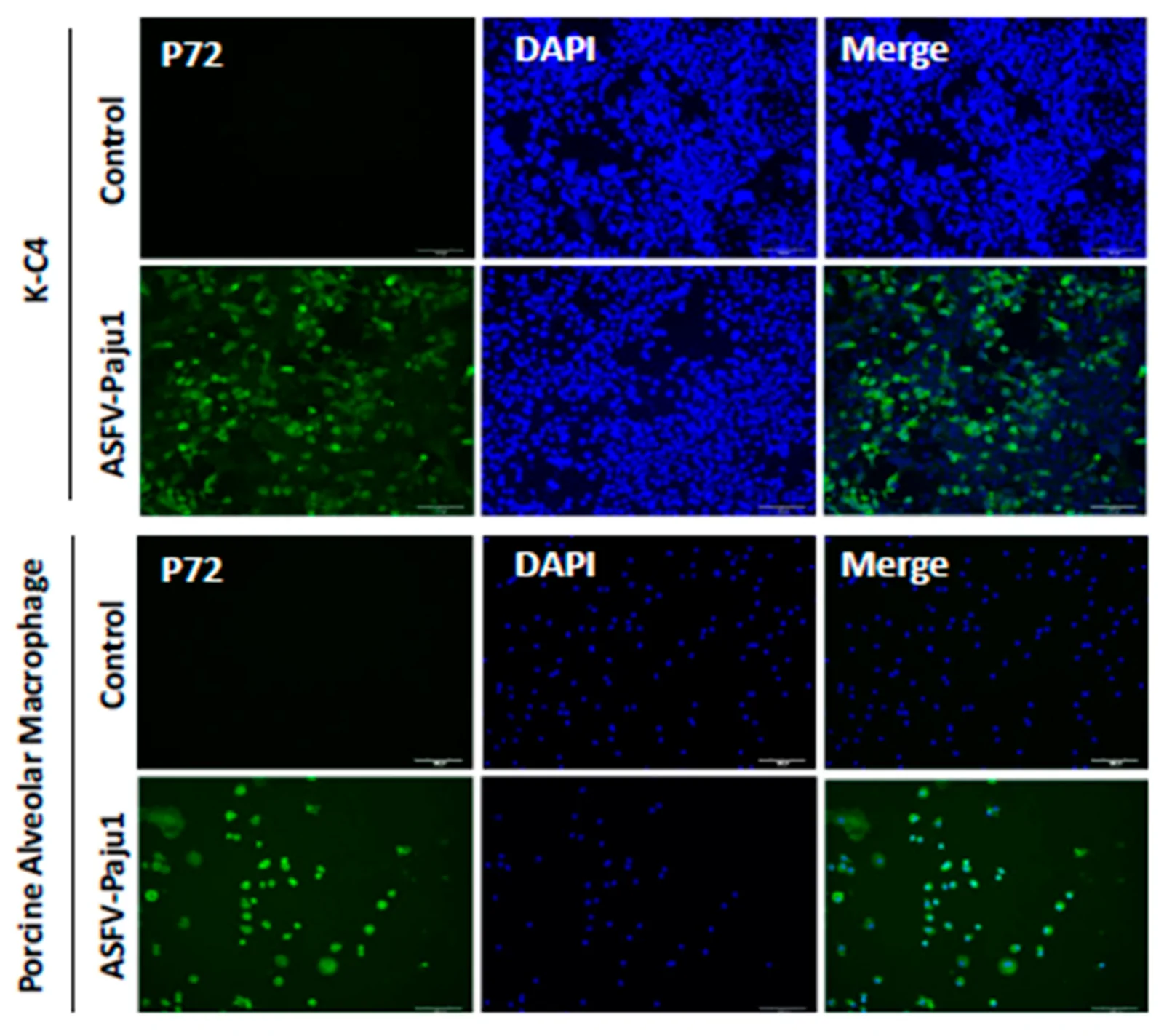
Detection of ASFV p72 protein in infected K-C4 cells by immunofluorescence assay (IFA). K-C4 cells and primary porcine alveolar macrophages (PAMs) were infected with ASFV and examined at 4 days post-infection (dpi). Cells were fixed with 4% paraformaldehyde and stained using a monoclonal antibody against ASFV p72 followed by an Alexa Fluor^®^ 488-conjugated secondary antibody. Cell nuclei were counterstained with 4′,6-diamidino-2-phenylindole (DAPI). Green fluorescence indicates ASFV p72 protein expression, whereas blue fluorescence indicates cell nuclei. Representative images were captured using the CELENA^®^ X High Content Imaging System and analyzed with CELENA^®^ X Cell Analyzer software (version 1.6.0). Images were acquired at 100× magnification.

**Figure 4:**
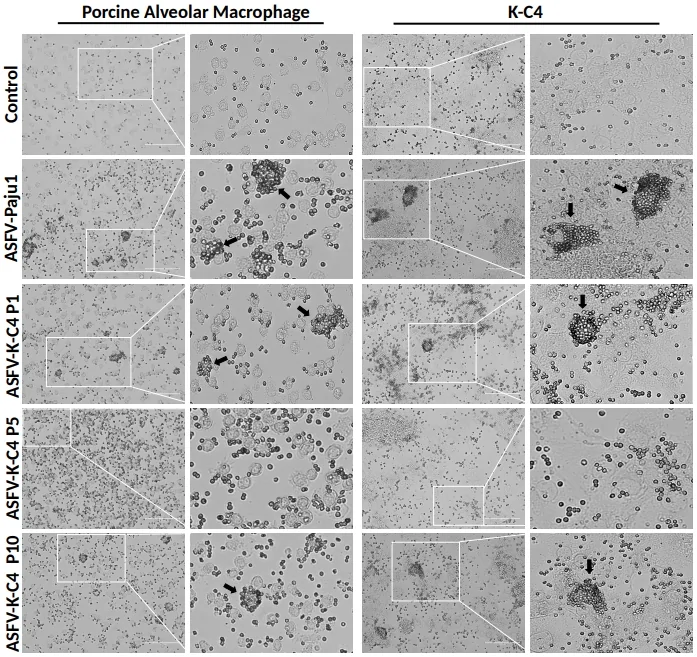
Passage-dependent loss and reappearance of hemadsorption during serial passage of ASFV in K-C4 cells. Porcine alveolar macrophages (PAMs) and K-C4 cells were infected with the parental Korea/Pig/PAJU1/2019 isolate (ASFV PAJU1) or viruses collected after passage 1 (P1), passage 5 (P5), or passage 10 (P10) in K-C4 cells. Uninfected cultures served as negative controls. Representative images were acquired at 4 days post-infection (dpi) after the addition of porcine erythrocytes. Characteristic erythrocyte rosettes (indicated by black arrows) were observed in both cell types infected with the parental virus or P1, were not detected in P5-infected cultures, and reappeared in P10-infected cultures. Cytopathic effects were observed in all virus-infected cultures. Images were acquired at 200× magnification.

**Figure 5:**
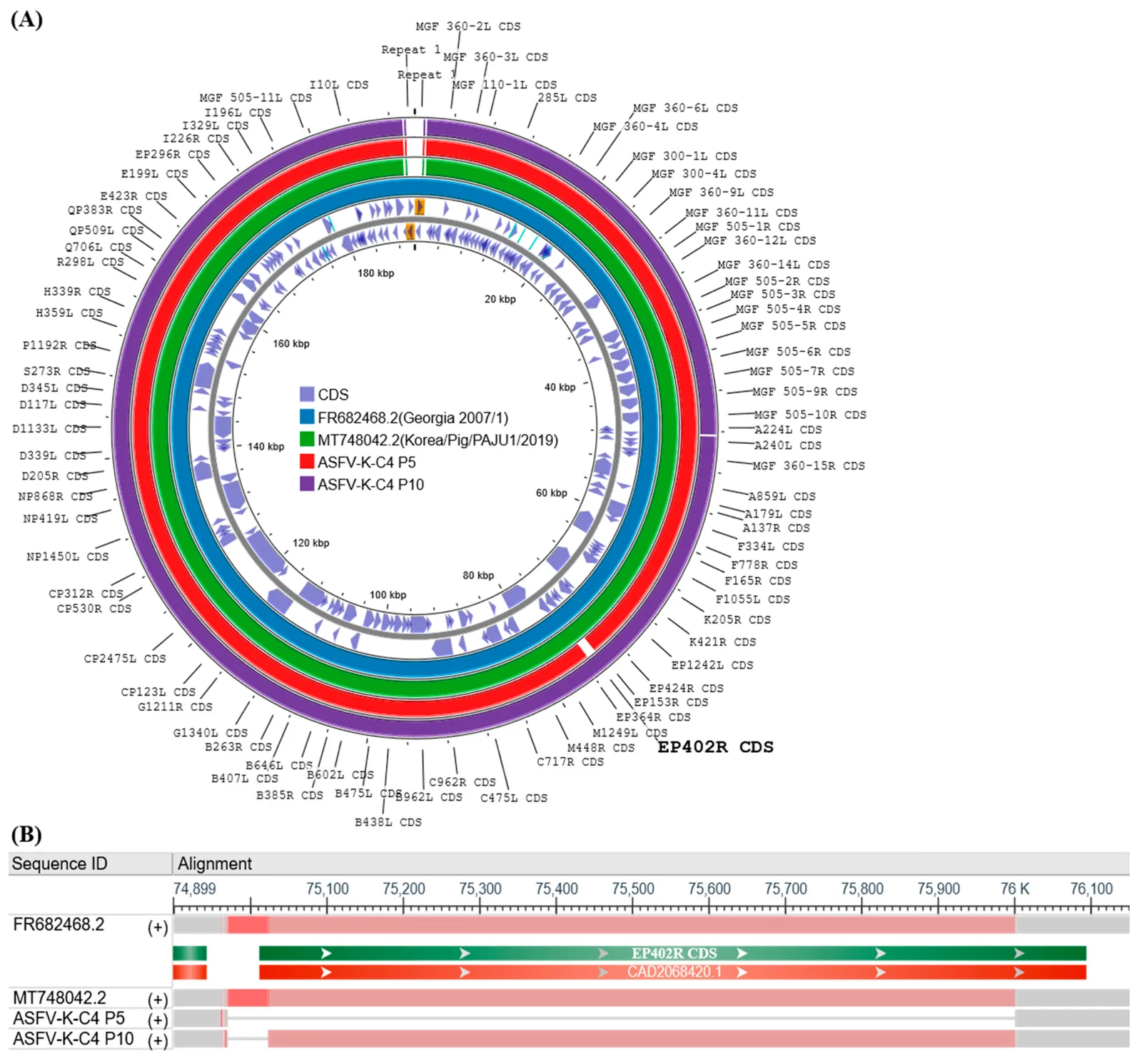
Whole-genome and *EP402R* sequence comparisons of ASFV PAJU1 during serial passage in K-C4 cells. (**A**) Circular whole-genome comparison of ASFV Georgia 2007/1 (GenBank accession no. FR682468.2; blue), the parental Korea/Pig/PAJU1/2019 isolate (MT748042.2; green), ASFV-K-C4 P5 (red), and ASFV-K-C4 P10 (purple). The concentric-ring map was generated using Proksee [18], and the position of the *EP402R* coding sequence (CDS) is indicated. (**B**) Multiple sequence alignment of the *EP402R* genomic region from the four viral genomes. Relative to the Georgia 2007/1 reference and parental ASFV PAJU1 genomes, P5 exhibited an extensive deletion encompassing most of the *EP402R* coding region, whereas P10 retained a substantially larger portion of the coding sequence and exhibited a shorter residual deletion. Sequence gaps indicate deletions relative to the reference and parental genomes. The alignment was visualized using the NCBI Multiple Sequence Alignment Viewer (version 1.26.0).

**Table 1:** Next-generation sequencing and consensus genome characteristics of ASFV PAJU1-derived viruses at passages 5 and 10 in K-C4 cells.

Virus	Total Reads	Reads Mapped to Georgia 2007/1, n (%)	Mean Coverage Depth (×)	Consensus Sequence Length (bp)	*EP402R* Deletion Relative to Georgia 2007/1	HAD Phenotype
ASFV-K-C4 P5	1,267,148	1,165,008 (91.94)	797.6	189,129	1032 bp (nt 74,969–76,000)	Negative
ASFV-K-C4 P10	772,655	463,580 (60.00)	302.5	190,187	55 bp (nt 74,969–75,023)	Positive

Note: Reads were mapped to the ASFV Georgia 2007/1 reference genome (GenBank accession no. FR682468.2). HAD, hemadsorption.

## Data Availability

The original contributions presented in this study are included in the article. Further inquiries can be directed to the corresponding authors.

## Abbreviations

The following abbreviations are used in this manuscript:

APQA	Animal and Plant Quarantine Agency
ASF	African swine fever
ASFV	African swine fever virus
bp	base pair
BSL-3	biosafety level 3
CD2v	CD2-like viral protein
CDS	coding sequence
CPE	cytopathic effect
Ct	cycle threshold
DAPI	4′,6-diamidino-2-phenylindole
DMEM	Dulbecco’s modified Eagle medium
DNA	deoxyribonucleic acid
dpi	days post-infection
FBS	fetal bovine serum
HAD	hemadsorption
HEPES	4-(2-hydroxyethyl)-1-piperazineethanesulfonic acid
IACUC	Institutional Animal Care and Use Committee
IBC	Institutional Biosafety Committee
IFA	immunofluorescence assay
IgG	immunoglobulin G
kbp	kilobase pair
KCLB	Korean Cell Line Bank
KVCC	Korean Veterinary Culture Collection
MEM	minimum essential medium
NCBI	National Center for Biotechnology Information
NEAA	non-essential amino acids
NGS	next-generation sequencing
PAMs	porcine alveolar macrophages
PBS	phosphate-buffered saline
PCR	polymerase chain reaction
qPCR	quantitative real-time PCR
RBCs	red blood cells
RPMI	Roswell Park Memorial Institute medium
SPF	specific-pathogen-free
